# Enhancement of Closed-State Inactivation by Neutralization of S4 Arginines in Domain IV of a Sodium Channel

**DOI:** 10.3389/fphar.2012.00143

**Published:** 2012-07-18

**Authors:** Tzur Paldi

**Affiliations:** ^1^The Hebrew University of JerusalemJerusalem, Israel

In PNAS, Capes et al. (2012) showed that the voltage sensor of domain IV in the skeletal muscle voltage-dependent sodium channel is energetically coupled to the channel gate located at the outer side of the pore. For this, the authors elicited gating pore currents, also known as omega currents, through each of the channel's four voltage sensors, one at a time. They inventively showed that sodium channel blockers TTX and μ-CTX, which bind to the outer pore in the selectivity filter region of the channel, affect the gating pore currents through domain IV, suggesting a coupling between conformational transitions in the selectivity filter and the movement of S4 in the voltage sensor of domain IV.

The authors did not refer to two important aspects, which are tightly related to their experimental approach. The first is that neutralization of arginines on S4 (by R to Q substitutions) used to elicit gating pore currents through each of the voltage sensor mutants, may abolish the voltage sensitivity of the voltage sensor (Bao et al., 1999), and position it in the activated or partial activated state (Gagnon and Bezanilla, 2009). The second aspect relates to the predominant role that domain IV voltage sensor plays in coupling activation with fast inactivation in sodium channels. The voltage sensor of domain IV is specialized in energizing a cytoplasmic inactivation element (IFM), connecting the third and fourth homologous domains, into the inner mouth of the pore via a mechanism known as “ball and chain” fast inactivation. Further, it was shown that a neutralizing mutation at the outermost S4 arginine in domain IV, which is associated with the paramyotonia congenital disease, greatly affects the coupling between activation and inactivation in the rat and human skeletal muscle sodium channels (Chahine et al., 1994).

It is therefore likely that activation of the voltage sensor of domain IV by the neutralizing mutations shifts the channel into a closed-state inactivation (Armstrong, 2006), which is associated with gating transitions that favor TTX binding to the outer pore (Patton and Goldin, 1991). If this is indeed the case, it points out to coupling between closed-state gating transitions at the outer pore gates with virtually all of the four voltage sensors, and not only with that of domain IV. Accelerated transitions in the selectivity filter at the outer gate due to occlusion of the cytoplasmic side of the pore by N-type inactivation particles is well established in homotetrameric K^+^ channels, though the parallel process in eukaryotic sodium channels is still under debate.

The technical limitation arises by channel entrance into a closed-state gating transitions due to neutralization of S4 arginines should be addressed to refine the experimental approach used by Capes et al. in order to make it more reliable. For example, it is possible to bring each of the mutant channels into a uniform gating state using a conditioning protocol that imposes channel entrance into a closed-state inactivation prior to recording of the gating pore currents, or use a mutant channel in which its fast inactivation is removed by mutating the IFM motif between domains III to IV.
